# Competing patterns of prestige in Latin American Spanish: implicit attitudes towards informal address in two Uruguayan cities

**DOI:** 10.1515/ijsl-2025-0058

**Published:** 2026-02-20

**Authors:** María Irene Moyna, Verónica Loureiro-Rodríguez, Elif Fidan Acar

**Affiliations:** Department of Global Languages and Cultures, Texas A&M University, 302B Academic Building, TAMU 4238, College Station, TX 77843-4238, USA; Department of Linguistics, University of Manitoba, 535 Fletcher Argue Building, Winnipeg, MB R3T 2N2, Canada; Department of Mathematics & Statistics, University of Guelph, 50 Stone Road East, Guelph, ON N1G 2W1, Canada

**Keywords:** Uruguayan Spanish, address system, matched guise test, language attitudes

## Abstract

This study uses a matched-guise test to examine attitudes toward informal address in Uruguayan Spanish, which features two competing pronouns (*tú, vos*), their associated verb forms (*tienes* ‘you have_T_’ vs. *tenés* ‘id_V_’), and mixed combinations (TT, VV, TV). The analysis compares perceptions in Montevideo and Rocha. Two Montevideo speakers (one male, one female) were evaluated by raters from Montevideo (*N* = 82) and Rocha (*N* = 75) on attributes related to status, solidarity, and perceived national and regional identity. A cumulative link mixed-effects model showed that all variants were perceived as Uruguayan. Montevideo raters judged VV as the most Uruguayan, followed by TV, whereas Rocha raters showed no significant differences among variants for the male guise, but perceived the TT female guise as most Uruguayan. In both locations, TT increased perceptions of speakers as less from Montevideo and more from Rocha. Gender effects emerged: the female speaker was perceived as less from Rocha and more from Montevideo than the male when using VV, indicating heightened sensitivity to women’s address choices. Rocha raters evaluated the female VV guise as less proper (*correcta*) than the male and the female TV guise as more disagreeable (*antipática*). These patterns suggest intersecting forms of prestige: VV and TV carry metropolitan prestige, while TT holds local covert prestige linked to authenticity, solidarity, and resistance to change. This study demonstrates the enduring role of informal address as a regional marker and the importance of speaker gender in attitudinal research.

## 1 Introduction

This study summarizes the findings of a comparison between attitudes to informal address in two cities in Uruguay using the Matched Guise Test (MGT), an experimental technique which measures implicit linguistic attitudes (Lambert et al. 1960). The two locations include the capital, Montevideo, and Rocha, a small city on the eastern seaboard, which were chosen because they differ markedly in their use of pronominal and verbal vernacular address forms. This amply documented contrast is one of the most peculiar dialectological features of Uruguay (Bertolotti 2011; Bertolotti and Coll 2003; Mendoza 2005; Rona 1967; Steffen 2010a; Weyers 2009, 2012, 2013, 2014).

Indeed, in its small territory Uruguay exhibits several competing variants of the second person singular informal address paradigm, mimicking at small scale an important morphological distinction in Latin American dialects more generally. While many varieties follow Peninsular Spanish in addressing a singular interlocutor with the second person pronoun *tú* and its corresponding verbal paradigm (typically identified as *tuteo* and represented here as TT), a significant subset of dialects prefer *vos*, the etymological plural, accompanied by its own verbal paradigm (i.e., known as *voseo*, and represented as VV). As we shall see in greater detail in Section 2.1, both *tuteo* and *voseo* paradigms are present in different regions of Uruguay. In addition, it is possible to find mixing of the *tú* pronoun with verbs corresponding to *vos*, in a combination we will refer to as hybrid *voseo* and represent as TV in the remainder of this paper.1The classic universal nomenclature used in address studies since Brown and Gilman (1960) (T for familiar, and V for deferential) does not work well with a tripartite system like that of USp. We have thus based our coding on the most straightforward abbreviations, i.e., the first letter of each form. Thus, T stands for any paradigmatic form of *tuteo*, while V stands for *voseo* forms; TV should be interpreted as the mixing of these two forms. This language-specific nomenclature provides the simplest way to retrieve full forms from abbreviations. The formal *usted* is not the focus of this paper and consequently no abbreviations were used to represent it.


The significance of second person informal address in the evaluation of Latin American Spanish varieties cannot be overstated. Even in attitudinal studies whose focus is not specifically on address (e.g., Chiquito and Quesada Pacheco 2014), these forms are so salient that they are repeatedly mentioned in survey responses. For instance, in García de los Santos (2014), unprompted Montevideo participants pointed to speakers from the east of Uruguay as the “most respectful” because they use *tú* instead of *vos*. Speakers of other varieties of Spanish tend to share these attitudes, with *voseo* typically identified as a non-prestigious, incorrect, rude, or otherwise undesirable feature in their or others’ speech (see Hernández 2014: 768 for Honduras; Rojas 2014: 144 for Chile; Flores Mejía 2014: 446 for Ecuador; Llull and Pinardi 2014: 39 for Argentina; Morett 2014: 923 for Mexico). In other words, address forms are very salient to folk perceptions of variation and an important determinant of dialectal preferences (for folk linguistics, see Preston 1986).

Linguists have also acknowledged the centrality of address by making it the focus of attitudinal studies in both Central America (Quintanilla Aguilar 2009 for El Salvador; Quintanilla Aguilar and Rodríguez Prieto 2014 for Costa Rica) and South America (Ennis 2011 for Ecuador; Weyers 2021 for Colombia; Haska 2021 for Chile). These studies employ explicit methodologies, gauging attitudes through survey questions that prompt participants to describe their usage and/or beliefs regarding one or more address forms. This approach is common because questionnaires and surveys are relatively simple to distribute, measure, and analyze, allowing researchers to gather data from a varied sample. However, it also has limitations. Survey items are often kept brief to guarantee clarity and increase completion rates, which may result in ambiguous wording (Kircher 2022). For example, a statement such as *me suena mejor* ‘sounds better to me’, may be interpreted prescriptively (“sounds more correct”) or descriptively (“sounds more natural”). These limitations may be unavoidable, especially when distinctions are subtle and hard to reflect upon consciously. These cases call for a more fine-grained analysis, for which the MGT is ideal.

Developed by Lambert and colleagues (1960) to examine attitudes towards English and French in Montreal, the MGT is an indirect method for evaluating not only participants’ attitudes towards others’ linguistic varieties but also towards their own. It relies on the assumptions that listeners share perceptions of traits associated with a particular way of speaking, and that direct methods often fail to capture privately held or nuanced attitudes, especially those vulnerable to social desirability bias. In this technique, participants listen to the recordings of several speakers using different linguistic varieties. Each recording serves as a guise, evaluated based on attributes reflecting the dimensions of status (e.g. intelligent, wealthy) and solidarity (e.g. friendly, funny). A key feature of the MGT is deception: participants must not realize that two or more guises were produced by the same speaker. To date, numerous variants of the MGT have been used to assess language attitudes in multilingual contexts (e.g., Guzzardo Tamargo et al. 2019; Loureiro-Rodriguez et al. 2013; Rangel et al. 2015). It has also been used to investigate attitudes towards English regional accents (e.g., Garrett et al. 1999, among many others), non-standard English accents (Berk-Seligson 1984; Cargile and Giles 1997; Ryan et al. 1977), tonal variation (Ray and Zahn 1999), and lexical diversity (Bradac and Giles 1988; Levin et al. 1994).

In recent years, the MGT has gained popularity to assess attitudes towards complex address configurations in Latin American dialects with regional and/or social variation, such as Chile, Colombia, and Central America. For example, Stevenson (2007) used it in his study of attitudes towards *voseo* and *tuteo* in Chilean Spanish, while Denbaum-Restrepo and Restrepo-Ramos have conducted several studies on Colombian address, mainly in Medellín, by combining MGT with production tasks to assess attitudes towards *voseo*, *tuteo*, and intimate *usted* (Denbaum-Restrepo and Restrepo-Ramos 2022, 2024). For her part, Denbaum (2021) and Denbaum-Restrepo (2025) focuses on attitudes in several Colombian cities (Medellín, Cali, Bogotá) towards polymorphism, the practice of combining multiple second singular forms in a single interaction with a single interlocutor. Only one study so far (Moyna and Loureiro-Rodríguez 2017) has focused on attitudes towards address variation in Uruguayan Spanish (USp), using a sample of 106 female raters from Montevideo. Its exclusion of men and speakers from other locations offers a limited view of attitudes towards address in the country.

The present study expands the use of MGT to a sample that includes men and women from both Montevideo and Rocha. Our first question (RQ1) examines which variants present in Uruguay (i.e., TT, TV, VV) are associated with Uruguayan identity, with Montevideo identity, and with Rocha identity by participants from these two locations. Our second question (RQ2) explores which address variants are associated with different status traits, and our third question (RQ3) considers their effect on solidarity traits. Within these three main questions, we also explore whether variant ratings differ based on speaker gender and participant characteristics (gender, age, education level).

This paper is structured in five additional sections. Section 2 summarizes the background that informed our selection of variants and attributes for the MGT experiment. Section 3 describes the implementation of the test used in the present study, our instruments, data collection, and statistical analysis of responses. Section 4 presents the results, in particular the main attitudinal patterns towards the three variants in the two locations. Section 5 interprets our results and highlights their implications. Lastly, Section 6 concludes the study and suggests possible expansions.

## 2 Background

This section includes a general overview of the informal second person singular paradigms of USp (2.1). This is followed by a contrast between the main geographic, historical, and social characteristics of Montevideo and Rocha, the two locations compared in this study, with special reference to their linguistic features (2.2). Finally, we offer a summary of prior research into attitudes to address in USp, using both direct and indirect measures (2.3).

### 2.1 The informal second person singular address paradigms of USp

The three main informal address paradigms of USp are presented in Table 1, namely, *voseo* (VV), hybrid *voseo* (TV), and *tuteo* (TT). *Voseo* is the most common in informal contexts, while a hybrid *voseo* pattern is slightly more marked and less frequent (Behares 1981; Bertolotti 2011; Moyna 2023). The opposite hybrid combination (VT, *vos tienes*) is only present in historical varieties (Rona 1967: 58). *Tuteo* forms are virtually indistinguishable from those used in the rest of the Spanish-speaking world. Table 1 excludes forms shared by all paradigms, such as pronominal possessives (unstressed: *tu casa* ‘your house’; stressed: *la casa tuya* ‘the house of yours’) and object (*te veo* ‘I see you’), and verbs such as the imperfect *tomabas* ‘you used to drink’, conditional *tomarías* ‘you would drink’, preterite subjunctive *tomaras* ‘you took.sbjv’, among others.

**Table 1:** Comparison of informal second person paradigms in Uruguayan Spanish.

Form	*Voseo* (*VV*)	Hybrid *Voseo* (*TV*)	*Tuteo* (*TT*)
**Pronouns**			
Subject pronoun	vos	tú	tú
Prepositional object	(para) vos	(para) ti	(para) ti
Prep. Obj. of ‘con’	con vos	contigo	contigo
**Verbs**			
Imperative	tomá	tomá	toma
Present indicative	tomás	tomás	tomas
Present subjunctive	tomes	tomes	tomes
Negative imperative	tomés	tomes (tomés)	tomes
Preterite	tomaste	tomaste	tomaste(s)

A detailed comparison shows that, in fact, only a few pronominal forms distinguish USp *voseo* and *tuteo*, including subject and prepositional object (*tú/vos me ves* ‘you_T/V_ see me’ *para ti/vos* ‘for you_T/V_’) (Fontanella de Weinberg 1999: 1404–5). After the preposition *con* ‘with’, USp speakers continue to prefer synthetic *contigo* ‘with you_T_’ to analytical *con vos* ‘id._V_’ The *voseo* verbal paradigm is distinct because imperative and present indicative forms are oxytonic and exhibit no irregularities such as shortened forms (imperative: *hacé* ‘do_V_.imp’, *vení* ‘come_V_.imp’ vs. *haz, ven* ‘id._T_’) or stem-changing verbs (present: *contás* ‘you tell_v_’ vs. *cuentas* ’id._T_’). The present subjunctive is more complex, since the dialect allows for a syntactico-semantic split along morphological forms (Fontanella de Weinberg 1979; Johnson 2016a, 2016b; Johnson and Grinstead 2011; Moyna 2023). *Tuteo* forms are almost categorical in non-matrix clauses (e.g., *No creo que puedas* ‘I don’t think you will_T_ be able to’ vs. *No creo que podás ‘id._V_’). These same forms can also be used deontically (*¡No vengas!* ‘Don’t come_T_!’), but when the orders are interpreted as cessatives, i.e., meant to stop an action in progress, the likelihood of *voseo* increases (e.g., *¡No molestés!* ‘Stop bothering_V_!’) (Fontanella de Weinberg 1979; Johnson 2016a, 2016b). Finally, preterite *voseo* forms end in *–s* (Elizaincín and Díaz 1979), although the link between these two features is more tenuous, since this segment also appears in non-*voseante* non-standard varieties (Barnes 2012).

The three options presented in Table 1 have distinct regional, social, and pragmatic distributions, and they elicit different evaluations. *Voseo* is a regional norm shared with the rest of the Río de la Plata region; specifically, Buenos Aires, Asunción, and several cities of the Argentine interior (for parallels, see Planells 1986; Prevedello 1989; Prevedello et al. 1999; Steffen 2010b; Vidal de Bettini 1964; Fontanella de Weinberg and Donni de Mirande 2000). It is historically linked with the earliest Spanish settlement (Bertolotti 2015: 101; Castillo Mathieu 1982) and was retained in this marginal area of the colonial empire in rural vernacular speakers and adopted later by the urban working class (Moyna and Vanni Ceballos 2008). It is the most common and unmarked informal address throughout Uruguay, in all close relationships, both symmetrical (sibling, classmate) and asymmetrical (parent–child, uncle–nephew) (Bertolotti 2011). Over the course of the twentieth and twenty-first centuries, its contexts have expanded to cover increasingly larger circles of solidarity where closeness can build over time (e.g., the workplace) as *usted* has retreated (Moyna 2019).

USp also exhibits mixing of *tuteo* pronouns with *voseo* verbs (TV). This feature is less frequent than *voseo*, but much more common than *tuteo*, and has been described as a standard form of Montevideo (Bertolotti 2011; Weyers 2014). It occupies a middle ground between the intimate *voseo* and the distant *usted*. By employing *voseo* verbal forms, it comes across as less artificial than prescriptive *tuteo* (Lipski 1994), while avoiding the salient and overly intimate *vos* pronoun. It is more frequent among the upper classes (Behares 1981: 33), older speakers and women (Bertolotti and Coll 2003; Weyers 2009), contexts that require closeness with deference (Bertolotti 2011: 36; Shively 2015), and pragmatic functions of mitigation (Moyna 2020). As we will in Section 2.3, the sociopragmatic features of hybrid TV have been corroborated through the MGT.

Finally, although *tuteo* is not frequent in spontaneous speech in most of Uruguay, it was prescriptively presented as the written norm until recently (Oroño 2011), and it is still favorably viewed in school contexts (Bertolotti 2011; Weyers and Canale 2013). Moreover, *tuteo* is a vernacular feature in a limited (and shrinking) geographical area in the Eastern Atlantic seaboard, which has its epicenter in Rocha but also includes other cities further west (e.g., San Carlos; Rona 1967: 56). The next section describes this contrast in detail.

### 2.2 Montevideo and Rocha: a tale of two address norms

**Figure 1:**
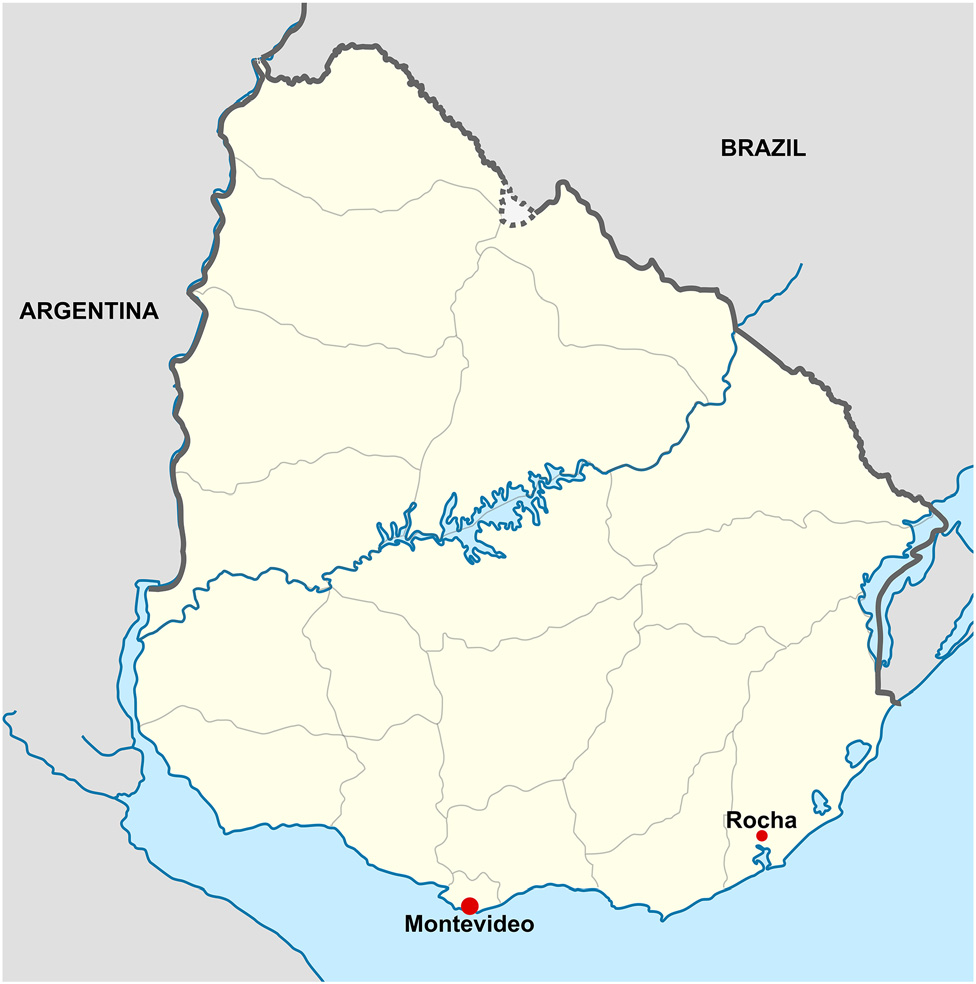
Map of Uruguay (from Wikimedia Commons, slightly modified).

Before discussing in depth the attitudinal differences to the address paradigms of Montevideo and Rocha, it is important to consider the unequal weight of these two locations in USp (Figure 1). Founded between 1726 and 1730, Montevideo is Uruguay’s uncontested center of economic and institutional life; its population of 1.4 million constitutes over one third of the country’s total and is about ten times larger than the second city, Salto (Instituto Nacional de Estadística 2024). This imbalance is due to Montevideo’s position as the main port of a supplier of raw materials for the global economy, which means that historically all transportation routes have converged there (Veiga 2010: 13). This centralization turned the capital into the main economic engine of the country, the seat of all three branches of government, most higher education institutions, hospitals, and other service providers. It should come as no surprise, then, that its linguistic variety is also the national norm.

This general observation holds true for address forms. The most frequent informal address in the Montevideo norm is *voseo*, although speakers can also mix *tuteo* pronouns and *voseo* verbs (TV, e.g., *tú tenés*, ‘you_T_ have_V_’). In fact, this hybrid constitutes a recognizable trait of the city within the region (Bertolotti 2011, 2015; Bertolotti and Coll 2003; Steffen 2010a).

By contrast, Rocha is the small capital (27,000 inhabitants) of the easternmost Uruguayan administrative subdivision, or departamento, also called Rocha. The rest of the population of this area lives in smaller towns and the rural interior. The city of Rocha was founded during the colonial period, in 1793 (Yacobazzo 2001: 27), in a scarcely populated but hotly contested area between Spain and Portugal. The original settlers were brought from further west but were originally from northwestern Spain and the Azores; once in Rocha, they built a subsistence economy around cattle-breeding, agriculture, and fishing. The area remained isolated for almost a century: the nearby Atlantic port of Santa María (present-day La Paloma) offered the only connection with Montevideo. The first railway line was established in the early twentieth century, and national highways were not built until the 1940s (Barranchini 1981). As a result, Rocha developed a distinctive and enduring culture. As communications improved in the late twentieth century, the rugged beauty of its pristine Atlantic beaches started to attract increasing numbers of tourists. That said, outside influences are limited mostly to the summer months, since the distance from the capital, the rough winters, and the lack of jobs and services are a disincentive to large-scale permanent internal migration.

Rocha speakers are proud of their dialectal variety, the foundation upon which they have built an ethnolinguistic identity connected to their Peninsular past and separate both from Portuguese and other Uruguayan varieties (Albertoni 2016; Weyers 2014). This double articulation of contrasts is documented in Albertoni (2016) through a corpus of public documents (e.g., local and national press, media, advertising, official communications). The author shows the evolution of the push and pull that has defined and challenged what it means to be “from Rocha”. Albertoni demonstrates that Rocha speakers have seen themselves as a bulwark against border Portuguese ‘*portuñol*’, and as such, their linguistic usage is an act of national sovereignty.2Anecdotally, Rocha’s emblem includes the motto *Donde nace el sol de la patria* ‘Where the sun of the fatherland rises’. On the other hand, over time, Rochenses have become exposed regularly to the national norm, both through interpersonal contact with outsiders and mass media (Weyers 2014). This increased awareness of dialectal differences (Albertoni 2016: 101). One particularly complex reality to navigate has been the arrival of tourists, since it is perceived as an economic boon but also as a force that dilutes local identity. The strategy has been to turn the local variety into one of Rocha’s attractions, the linguistic counterpart of other commodified regional features (natural beauty, pure air, relaxation).

Rocha’s most salient linguistic feature is the use of *tuteo* (TT, e.g., *tú tienes* ‘you_T_ have_T_’). This usage, linked to the area’s late European colonization, is interpreted by locals as evidence of their variety’s superiority, correctness, and purity, summarized in the attribute *castizo* ‘authentically Spanish’.3The third acceptation of *castizo* in the Diccionario de la Real Academia applies specifically to language that is *puro y sin mezcla de voces ni giros extraños* ‘pure and without admixture of foreign words or expressions’ (DRAE 2025). Moreover, it is imbued with ethical values, such as agreeableness and good manners. For example, the stereotypical expression by which it is represented, *toma tú que te toca a ti* ‘here you are; it is your turn’ not only employs *tuteo* but also evokes polite turn-taking. *Tuteo* can also have negative associations (Albertoni 2016: 102), including conservatism and resistance to change. By the same token, the adoption of *voseo* can also be evaluated ambiguously, as an act of betrayal and rudeness or as evidence of openness and progress.

The coexistence of *voseo* and *tuteo* results in a multilayered competition between a national norm and a regional vernacular/supranational standard. That is, while in Montevideo *tuteo* can play the role of a superimposed prescriptive model, it can also evoke a remote and “quaint” region of the country. By contrast, in Rocha, spontaneous *tuteo* is bolstered by awareness of its Pan-Hispanic usage, while at the same time it lacks the prestige of the national metropolitan *voseo* norm. In the next section, we describe in some detail the findings of earlier studies that have examined attitudes of Montevideo and Rocha speakers to their address variants.

### 2.3 Attitudes towards *tuteo* and *voseo* in USp

Several studies have explored attitudes towards address in USp using direct methods. Weyers (2013) presents a detailed analysis of attitudes in Montevideo with data from a large Likert-scale survey (*N* = 431). Results revealed positive attitudes towards *tuteo* that were not reflected in its actual use. Speakers under the age of 40 preferred *vos* to address others their age, even if they were unacquainted, while older speakers, especially women, favored *tú* in those situations. Young respondents considered *vos* appropriate even in contexts of social distance and power imbalances (e.g., with superiors at work). Additionally, young speakers and those from upper social classes considered the hybridization of *tú* and *voseo* verbs as correct, while older respondents were more likely to accept *tuteo* verbs as possible in speech.

In a separate attitudinal survey done in Montevideo with 82 teachers, Weyers and Canale (2013) identified similar generational differences, with the most senior teachers showing a stronger preference for *tuteo* compared to their younger counterparts. Private school educators reported using *vos* in the classroom more frequently than those in public schools. That said, most teachers considered it acceptable for students to use *vos* unless it was pragmatically inappropriate, such as when addressing the principal. Notably, *voseo* was absent from teacher training: older teachers recalled being advised to use *tú*, while younger teachers reported receiving a more permissive instruction that did not prescribe either form.

Teachers’ attitudes were also the focus of Holt’s (2018) mixed methods study (*N* = 20) in Maldonado, a transitional *tuteo*-*voseo* area. The implicit task involved preschool teachers and classroom volunteers reading aloud to their pupils. The stories, written by contemporary Montevideo writers, typically employed *voseo*. Holt reasoned that quantifying changes to the second singular address forms during the reading could offer a window into readers’ implicit attitudes. Although only nine readers altered the original address forms (Holt 2018: 104), the results were still suggestive, since 11 of the 14 changes aligned with the form favored by the reader. Holt supplemented these measures with explicit attitude questions. She found that, although most teachers agreed that *tuteo* should be used to address students orally (*tuteo*: 55 %; *voseo*: 20 %; either: 25 %) and in written instructions (*tuteo*: 95 %; either: 5 %), the majority disagreed with correcting preschoolers’ *voseo* use (85 % vs. 15 %).

Attitudes to address in Rocha have also been analyzed using explicit methods. In an attitudinal survey among young speakers (*N* = 58; mean age: 18.7) Weyers (2014) examined informal address attitudes in the city. Respondents used a five-point Likert scale to evaluate twelve statements, ten of which focused on *tuteo*, *voseo*, and hybrid forms in spontaneous speech or in advertising. In each statement, participants were asked to decide whether they believed the utterance presented was natural sounding, correct, or appropriate. Overall, participants rated verbal *tuteo* higher compared to *voseo* forms. Moreover, the presence of *tú* pronouns was not sufficient to raise the ratings of utterances with *voseo* verbs. Respondents also felt that advertisements presented in *tuteo* (*Imprime tus fotos aquí* ‘Print_T_ your photos here’) appealed to them in their own variety (literally, *me habla en “mi” idioma* ‘speaks to me in “my” language’, Weyers 2014: 391) more than *voseo* forms (*Viví con frescura* ‘Live_V_ with coolness’). That said, the formulation of the questions led to some puzzling results. For example, the items to evaluate *¿De dónde eres tú?* ‘Where are_T_ you_T_ from?’ and *¿De dónde sos tú?* ‘Where are_V_ you_T_ from?’ obtained virtually identical scores (means of 3.90, 3.91, respectively) despite the contrast between verbs. This may have been due to differences in the wording of their carrier sentences: while the *tuteo* statement had to be evaluated as *una pregunta apropiada* ‘an appropriate question’ the assessment of the hybrid statement was worded as *me suena correcto* ‘sounds correct to me’. Despite these limitations, it was clear that *tuteo* was considered more common, correct, or preferred by the respondents over utterances that included verbal *voseo*, even in the presence of *tú* pronouns. The last two statements in the survey were more general, since they focused explicitly on perceptions of the local and national varieties. Young Rocha respondents expressed strong pride towards their variety, which they agreed was “purer”, an attitude that Weyers hypothesized may contribute to its maintenance.

The use of MGT to analyze attitudes towards address in USp varieties is more incipient. To date, we are familiar with only one such study (Moyna and Loureiro-Rodríguez 2017), which analyzed the attitudes towards Montevideo informal address forms and serves as our most direct antecedent and inspiration. Female participants (*N* = 106) evaluated three informal address forms (*tuteo*, *voseo*, and TV hybrid) recorded by four speakers (2M, 2F) for a total of 12 guises. *Voseo*, followed by hybrid TV, was associated with Montevideo and with Uruguayan identity. By contrast, *tuteo* was rated as neutral for these attributes, which shows that this address form is excluded from the national norm by these Montevideo raters. *Tuteo* was also associated with traditional values (politeness, conservatism), regardless of speaker gender. By contrast, *voseo* was associated with modernity and openness, with few differences by speaker gender. The hybrid *voseo* variant had the highest ratings for prestige and was the most sensitive to speaker gender. Both male and female hybrid TV guises were rated highly for personal appeal attributes. For men these tended to be character traits, while for women they were external features. Men were perceived as more polite than women when they used hybrid forms, which suggests different baseline expectations: men were rewarded for using TV, while women were punished for not using it, as one would expect with a form with overt prestige, along the lines of Labov (1966) and Trudgill (1972).

To summarize, in USp informal address forms elicit conflicting attitudes among speakers. On the one hand, there is a national norm, *voseo*, which draws its symbolic power from its use as a regional norm in the Río de la Plata region in opposition to other Latin American varieties. On the other, *tuteo* continues to be favored by prescriptive purist discourses. The TV hybrid is not as frequent as *voseo*, but in some respects is a local prestige norm, favored by older and more overtly prestigious groups. Rocha’s spontaneous vernacular use is equated with the untouched natural beauty of the department. Ironically, this is also one of its weaknesses among young speakers of Montevideo, who see it as antiquated. Given this ambivalence, the MGT presents several advantages over other approaches to compare attitudes in Montevideo and Rocha. It may help elucidate subtle aspects of *voseo*-*tuteo* variation that have escaped identification and detection by other methods.

## 3 Methodology

This section includes a description of the general features of the MGT and its overall advantages and disadvantages compared to other methods of attitudinal elicitation. This is followed by a description of the specific materials and design of the present study, the method used to recruit participants and collect data, the participant sample, and the statistical analysis employed.

### 3.1 The matched-guise test

In the traditional MGT design, participants listen to the recordings of the same individual reading an identical text in two or more linguistic varieties. Each recording represents a distinct “guise” that participants evaluate on Likert scales, assessing traits commonly associated with the dimensions of status and solidarity (Zahn and Hopper 1985). Using the same speaker for all linguistic varieties under investigation controls for speaker-level features, ensuring that any differences in ratings across guises reflect participants’ attitudes toward the linguistic variety and the speakers associated with it rather than toward the individual producing the recording. While language-based differences in the recordings are not concealed, participants must remain unaware that the same speaker produces multiple guises, and thus filler voices are interspersed between target recordings to help maintain this deception.

The speakers recording the stimuli are typically close in age, which should be considered when interpreting the results, as the perceived age of the speaker may influence participants’ attitudes toward the linguistic variety under investigation (Loureiro-Rodríguez and Acar 2022). Similarly, the sex of the speaker may also impact participants’ ratings, since certain linguistic features are often associated with gendered social norms or stereotypes, as seen in MGT studies across various contexts (e.g., Echeverria 2005; Ihemere 2006; Loureiro-Rodríguez et al. 2013; Rangel et al. 2015; Moyna and Loureiro-Rodríguez 2017).

The MGT offers several strengths. First, because participants’ attitudes are elicited indirectly, they are less susceptible to social desirability bias, allowing for a more accurate representation of covert attitudes that may not emerge through direct methods. Second, the MGT is grounded in a substantial body of attitudinal research, particularly regarding the use of the status and solidarity dimensions, lending this technique a strong theoretical and empirical foundation. However, there are also limitations to consider. Hearing multiple recordings of the same speaker using different linguistic varieties may make differences in language use more noticeable than in natural, spontaneous interactions, thus potentially influencing participants’ judgments. Additionally, having individuals read a written passage for the stimuli reduces the spontaneity and natural flow of oral speech, which may also affect participants’ responses.

### 3.2 Materials

Stimuli: The stimulus text is a shortened version (33 words) of the one used in Moyna and Loureiro-Rodríguez (2017). Designed to resemble a voicemail, it helps maintain a conversational tone when being read. We created three versions of the text, differing only in their second-person singular pronouns and verbs (see Appendix). The main guises (three per speaker) were recorded by two young adults from Montevideo in their 20s (1M, 1F). The decision to employ only one male and one female guise sought to avoid fatigue while guaranteeing that all participants would listen to all six guises. Admittedly, the use of single male and single female speakers made it impossible to tease apart gender from other potential individual variables. However, to minimize the possibility of confounding factors, the two participants chosen were closely related (cousins) users of the normative variety of USp typical of college-educated Montevideo speakers, who had been recorded in the earlier study that detected no issues with their voices (Moyna and Loureiro-Rodríguez 2017). Additionally, two middle-aged speakers in their 40s to early 50s, also from Montevideo, recorded the filler guises (*n* = 4). All recordings took place in the speakers’ homes using a Marantz PDM 620 audio device and were saved as MP3 files on a laptop.

Traits: To evaluate the recordings, we compiled a list of ten attributes directly related to our research objectives. These attributes are presented in three descriptive groups.4These groups are merely included to ease the presentation and do not have any analytical value. The first includes three traits related to status (*correcta* 5Regardless of speaker gender, all adjectival attributes appeared in the feminine form to match the feminine noun *persona* ‘person’ in the header. ‘proper’, *moderna* ‘modern’, *con estudios universitarios* ‘university educated’), and the second comprises four traits related to solidarity (*cortés* ‘polite’, *delicada* ‘gentle’, *falsa* ‘phony’, *antipática* ‘disagreeable’), all of which had been shown to discriminate well in Moyna and Loureiro-Rodríguez (2017). The third group includes three traits related to national identity and regional origin (*uruguaya* ‘Uruguayan’, *de Montevideo* ‘from Montevideo’, and *de Rocha* ‘from Rocha’). Each trait was rated on a 5-point Likert scale anchored at 1 “not at all” and 5 “very much”, with 3 representing a neutral option.

Socio-demographic questionnaire: A brief questionnaire was designed to gather information on participants’ age, gender, provenance within Uruguay, native language(s), and level of education attained (used as a proxy for socioeconomic status).

### 3.3 Design

The experiment was set up in Qualtrics. To prevent priming effects, the ethics forms and experiment instructions were presented in the *usted* (you.2sg.formal) form, which is considered neutral in the content of a survey or experiment. The experiment began with a description of the study, stating that the researchers were investigating Uruguayans’ perceptions of language variation, followed by the informed consent form, and the socio-demographic questionnaire. Next, the voice recordings were presented individually with instructions on how to play them, followed by the list of traits for evaluation. To minimize recognition, guises from the same speaker were spaced as far apart as possible (see Table 2). A within-subjects design was chosen due to uncertainty about participant recruitment.6The data collection process was complex because it was done in two different locations. We could anticipate that at least in one location (Rocha) online tools would not yield enough responses, as turned out to be the case. Because randomization is hard to implement in person, we decided against it to make the study comparable across the board. While this design can introduce carryover effects due to participants being exposed to multiple guises, it provides greater statistical power without requiring a large sample size.

**Table 2:** Order of presentation of the guises.

Filler	Guise	Guise	Filler	Guise	Guise	Filler	Guise	Guise	Filler
F	M	F	M	F	M	F	F	M	M
TT	TT	TV	VV	VV	TV	TV	TT	VV	TV

### 3.4 Participant recruitment and data collection

Data was collected between 2018 and 2023, and participants were recruited using a combination of methods: snowball sampling (Montevideo, Rocha) and in-person visits to higher education institutions (Rocha). The link to the experiment was shared with friends and acquaintances of the first author, and they were asked to pass it along to their social networks. Since recruiting participants from Rocha proved challenging, the first author contacted several higher education institutions to recruit participants and collect data in person. As a result, a paper-based version of the experiment was created. With institutional permission, the first author visited classrooms, briefly explained the study’s purpose (while maintaining deception), and invited students over the age of 18 to participate. Those who agreed were invited to participate after class, in an empty room of the institution, alone or in small groups. The researcher, a native speaker of Montevideo Spanish, ran the experiment in person in all cases. Participants listened to recordings played from the researcher’s laptop and recorded their responses either on paper or directly in Qualtrics.

### 3.5 Participant sample

A total of 197 participants took part in the study. To ensure data quality, we applied the following inclusion and exclusion criteria. First, individuals who were not from Montevideo or Rocha were excluded (*n* = 18). Second, we removed participants who showed evidence of malingering (e.g., providing identical responses to all attributes), failed to answer any of the questions in the evaluative portion of the test, or stopped contributing to the study after the first recording, which featured a distractor voice (*n* = 22).

We included data from 157 participants (Montevideo: *n* = 82; Rocha: *n* = 75) for the statistical analysis. All Montevideo participants completed the experiment online, while most Rocha participants (*n* = 60) provided responses in person. We assume that participants’ attitudes were not influenced by the data collection method and remained unchanged over the five years it took to collect sufficient responses for the study, since there were no major societal changes in Uruguay over the period.

**Figure 2:**
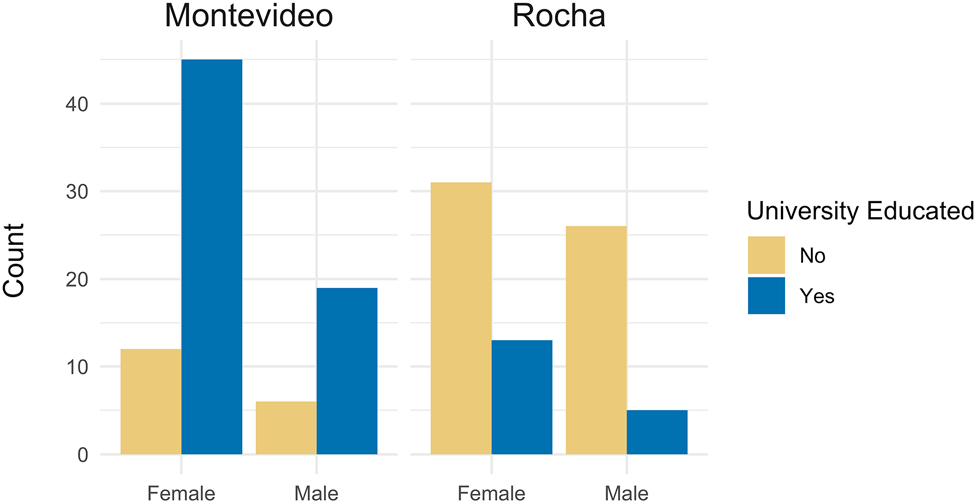
Distribution of gender and education level of participants in each location.

The gender distribution was similar across locations: over 69 % of participants identified as female in Montevideo (25M, 57F), and over 58 % in Rocha (31M, 44F). However, educational attainment differed between the two groups: in Montevideo, the majority had completed higher education (*n* = 64), whereas in Rocha, most had only finished secondary education (*n* = 57) (Figure 2).

### 3.6 Statistical analysis

The statistical analysis for this study is based on cumulative link mixed-effects models (Christensen and Brockhoff 2013), a regression framework well-suited for ordinal (Likert scale) responses that are repeatedly measured from the same participants (Loureiro-Rodríguez and Acar 2022; Taylor et al. 2022). We selected this approach not only for its appropriateness for ordinal data, but also to preserve the interpretability of each attribute relevant to our research objectives and properly account for dependence across multiple ratings from each participant. Other approaches, such as linear mixed-effects models, can also account for repeated ratings from participants. However, they are frequently built on response variables obtained from aggregate methods such as factor analysis or principal component analysis. The latter typically require continuous data (a condition not met by Likert scales), rely on subjective choices of rotations (in the case of factor analysis) and assume independence across ratings from the same participant (in sample covariance estimation).

To facilitate comparisons between the two locations, we fit separate models for Montevideo and Rocha. For each trait and location, we fit a cumulative link mixed-effects model using the ordinal package in R (Christensen 2023) and incorporating random effects for participants (to account for repeated responses) and fixed effects for the variant type (TT, VV, TV), speaker (M, F) and participant characteristics such as gender (M, F), age (numeric original-scale value7The age variable was not centered, as centering in cumulative link models does not influence coefficient estimates or their significance. ranging from 14 to 73 with median age 34.5) and having higher education (No, Yes). To assess potential differences in the attitudes towards the variants based on speaker gender, we also included an interaction term for variant type and speaker gender in these models. All reported results are based on the full models and were not subjected to stepwise variable selection to avoid potential bias due to post-selection inference (Tibshirani et al. 2016).

## 4 Results

The results section is presented in the same order as the research questions and includes the analysis of responses for the three identity traits (RQ1, Section 4.1), four status traits (RQ2, Section 4.2), and three solidarity traits (RQ3, Section 4.3). The results are reported and interpreted based on the significance and direction of regression coefficients in the fitted models, together with contrasts comparing variant-speaker gender combinations in each location. The latter comparisons are provided in Appendix – Part C.

### 4.1 Results on identity traits

**Figure 3:**
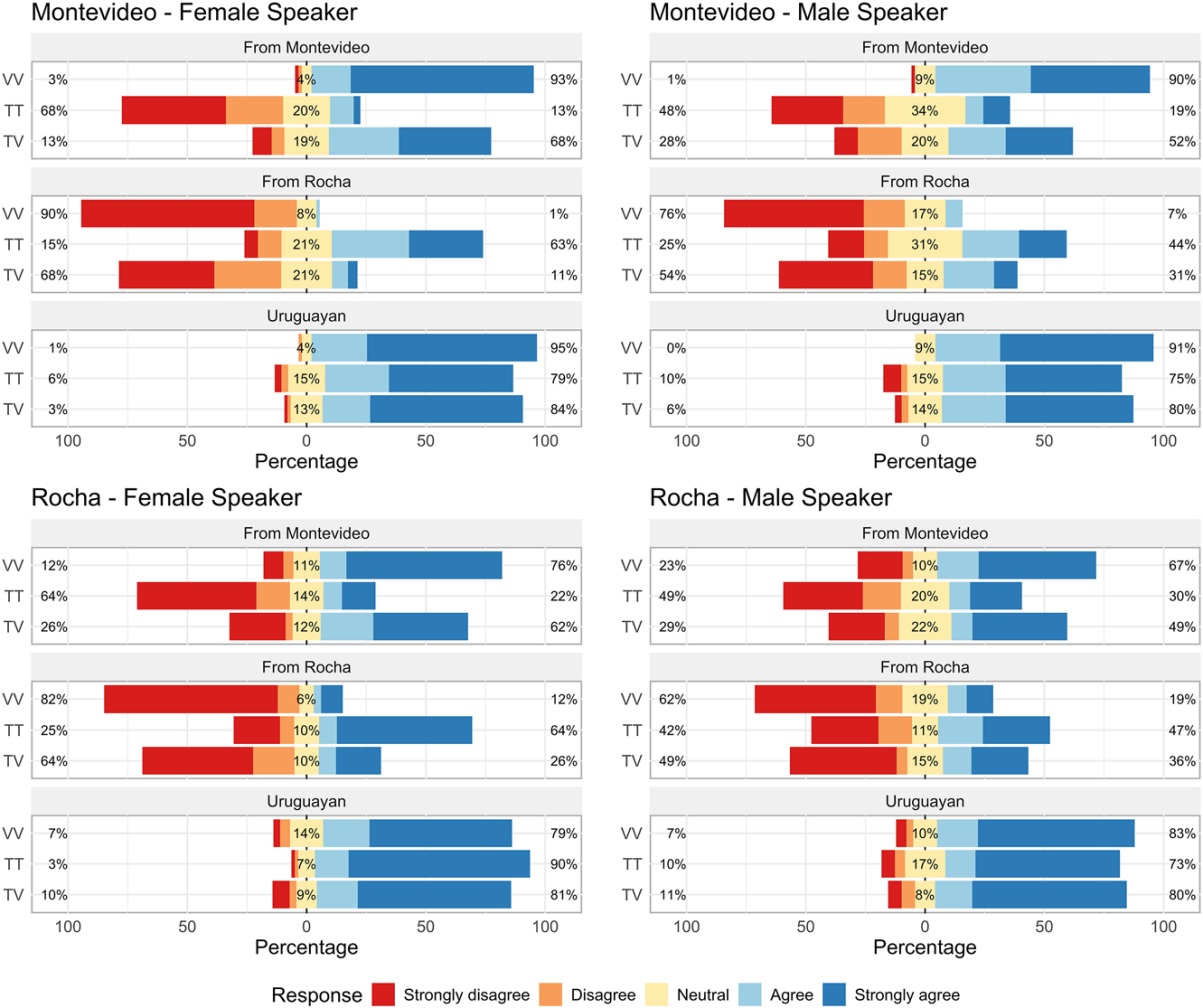
Distribution of the ratings on the identity traits of female and male guises across the three variants by participants in Montevideo and in Rocha.

Figure 3 displays the relative frequency of centered ratings (from “strongly disagree” to “strongly agree”) of Montevideo and Rocha participants on the identity traits across the three variants for each speaker. In both locations, participants perceived speakers as Uruguayan regardless of the variant they used. While participants identify VV and TV variants as mostly from Montevideo, the TT variant is considered more linked to Rocha identity. We also observe some visual differences in how the speakers were perceived when they use certain variants. For instance, the male speaker was perceived as less from Montevideo and more from Rocha compared to the female speaker when they used VV and TV but not when they used TT.

To formally evaluate the significance of the observed differences across variants and speakers, we present the output from the fitted cumulative link mixed-effects models for each of the three identity traits in Table 3. While direct interpretation of the coefficient estimates is complicated due to the logit link function used in these models, a negative sign in the estimate of a fixed effect covariate indicates a lesser degree of agreement (or a higher degree of disagreement) with the trait of interest compared to the base groups: the female speaker using the VV variant and female participants with university education. We thus conclude that participants from Montevideo perceived the TT and TV variants as less from Montevideo, more from Rocha, and less Uruguayan compared to the VV variant. Meanwhile, participants from Rocha also recognized TT and TV as less from Montevideo and more from Rocha; however, they regarded TT as significantly more Uruguayan than VV, with no significant difference in the Uruguayan identity between TV and VV. In both locations, the male speaker was perceived as less from Montevideo and more from Rocha compared to the female speaker, but this was mainly observed when using the VV and TV variants. No significant difference emerged with the TT variant, except that Rocha participants identified the female speaker using TT as more from Rocha compared to the male speaker. The opposite signs of the interaction effect between the TT variant and the male speaker, and the effect of the TT variant tend to neutralize each other, resulting in less significant or insignificant differences when the male speaker uses the TT variant compared to the female speaker.

**Table 3:** Results from the fitted cumulative link mixed-effects models for the identity traits. Reported are the coefficient estimates and their significance levels. Coefficients shown in boldface are statistically significant at the 0.05 level.

	From Montevideo		From Rocha		Uruguayan	
	Montevideo	Rocha	Montevideo	Rocha	Montevideo	Rocha
VariantTT	−4.44***	−2.81***	3.97***	3.00***	−1.33***	1.15**
VariantTV	−1.75***	−1.20***	1.46***	1.16**	−0.65	0.10
SpeakerM	−1.01**	−0.80*	0.79*	0.88*	−0.45	0.33
GenderM	−0.02	0.22	0.21	−0.13	0.47	−0.95
Age	0.00	0.00	0.00	0.00	0.03	−0.01
EducationNo	−0.13	−0.37	−0.41	0.23	−0.93	−0.56
VariantTT × SpeakerM	1.70***	1.50**	−1.57***	−1.90***	0.17	−1.63**
VariantTV × SpeakerM	0.32	0.66	−0.26	−0.60	−0.16	−0.19
SD (Intercept ID)	0.33	1.01	0.71	0.95	1.73	2.02
Num.Obs	440	407	440	394	440	420

Significance levels: *p < 0.05, **p < 0.01, ***p < 0.001.

It is important to note that the number of available responses differs across different traits as some participants did not provide a response for all guises of a trait. We utilize the available data for each trait and report the number of responses used in the analysis in Table 3 (Num. Obs.). The reported standard deviations quantify the between-participant variability under each fitted model, with a slightly higher variability in the responses of Rocha participants than those of Montevideo participants. The relative positions of the variant-speaker gender combinations in each location can be found in Table C1 in Appendix – Part C.

### 4.2 Results on status traits

**Figure 4:**
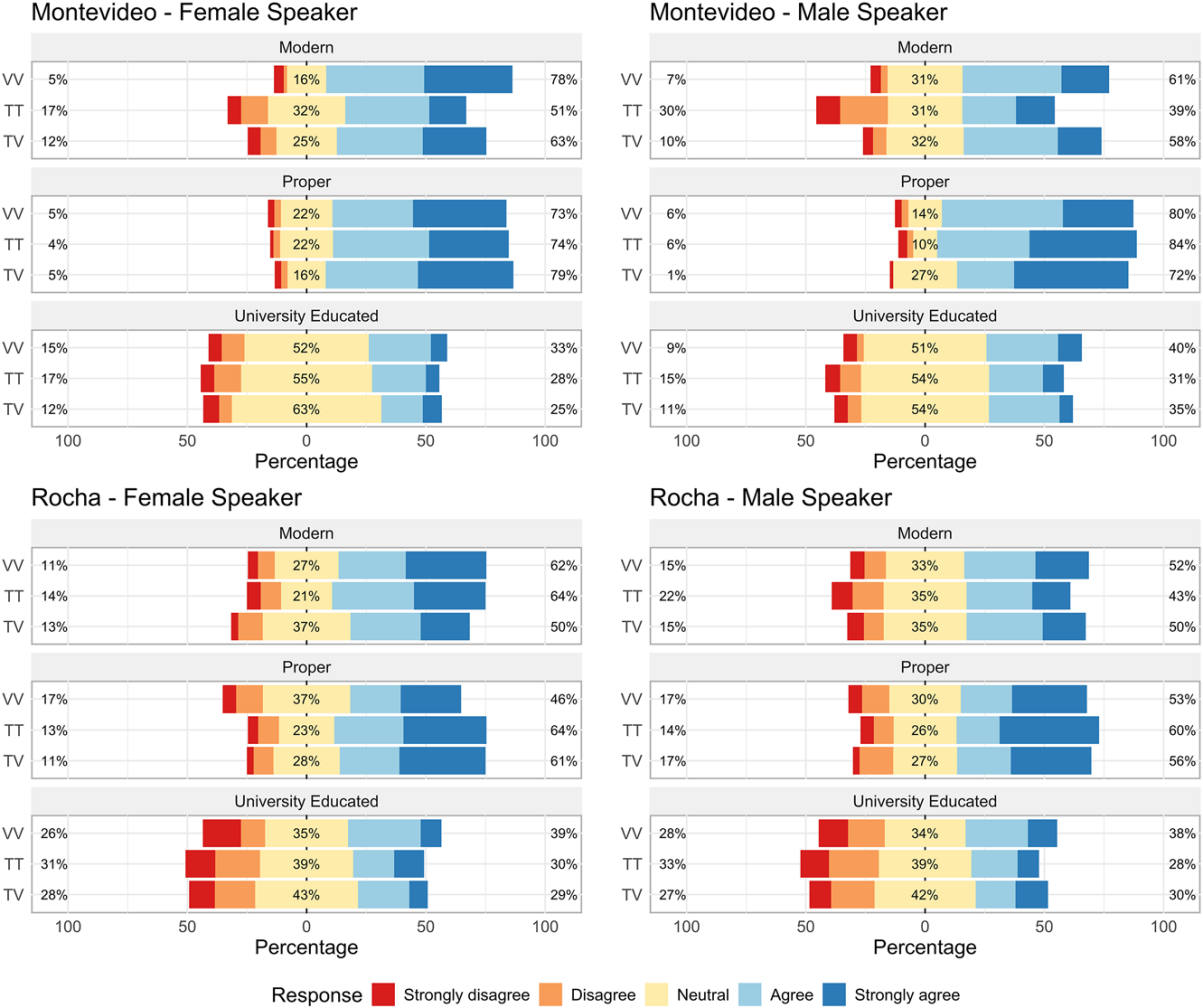
Distribution of the ratings on the status traits of female and male speakers guises the three variants by participants in Montevideo and in Rocha.

Figure 4 displays the relative frequency of centered ratings (from “strongly disagree” to “strongly agree”) of Montevideo and Rocha participants on the status traits across the three variants for each speaker. Overall, participants in both locations perceived the speakers as modern (*moderna*) and proper (*correcta*) but did not have firm opinions on their education levels. Our visual inspections suggest that Montevideo participants tend to rate speakers as more modern and more proper compared to Rocha participants.

The results from the fitted cumulative link mixed-effects models for each of the three status traits are presented in Table 4. In both locations, the female speaker was perceived as more modern than the male speaker, particularly when using the VV variant. The TT variant was perceived as significantly less modern than TV and VV in Montevideo for both speakers, with a significant difference between TV and VV only for the female speaker. In Rocha, the TV variant was perceived as significantly less modern than TT and VV for the female speaker, whereas for the male speaker, it was the TT variant that was considered the least modern. In Montevideo, no significant differences were observed between the variants in terms of the attribute *correcta* ‘proper’. However, for the male speaker, the TT variant was considered more *correcta* ‘proper’ than the VV variant, though this difference was only borderline significant. In Rocha, the VV variant was perceived as less proper than the other two for the female speaker, while for the male speaker there was no significant difference in perception across the three variants. Older participants tend to identify speakers as more highly educated and more modern in Rocha, and more proper in Montevideo. The relative positions of the variant-speaker gender combinations in each location can be found in Table C2 in Appendix – Part C.

**Table 4:** The results from the fitted cumulative link mixed-effects models for the status traits. Reported are the coefficient estimates and their significance levels. Coefficients shown in boldface are statistically significant at the 0.05 level.

	Modern		Proper		University educated	
	Montevideo	Rocha	Montevideo	Rocha	Montevideo	Rocha
VariantTT	−1.95***	−0.13***	−0.16	0.75*	−0.37	−0.27
VariantTV	−1.03**	−0.82***	0.12	0.70*	−0.24	−0.26
SpeakerM	−1.26***	−0.72***	−0.22	0.13	0.63	0.08
GenderM	−0.95	0.22	−0.69	0.20	0.28	−1.53*
Age	0.03	0.02***	0.05*	0.01	0.01	0.01***
EducationNo	0.65	0.63	−1.32	0.33	−0.60	0.49
VariantTT × SpeakerM	0.58	−0.41***	1.10*	−0.13	−0.28	−0.23
VariantTV × SpeakerM	0.85	0.68***	0.43	−0.41	−0.23	0.06
SD (Intercept ID)	2.36	1.97	2.60	2.11	2.94	2.48
Num.Obs.	440	412	443	421	440	392

Significance levels: *p < 0.05, **p < 0.01, ***p < 0.001.

### 4.3 Results on solidarity traits

**Figure 5:**
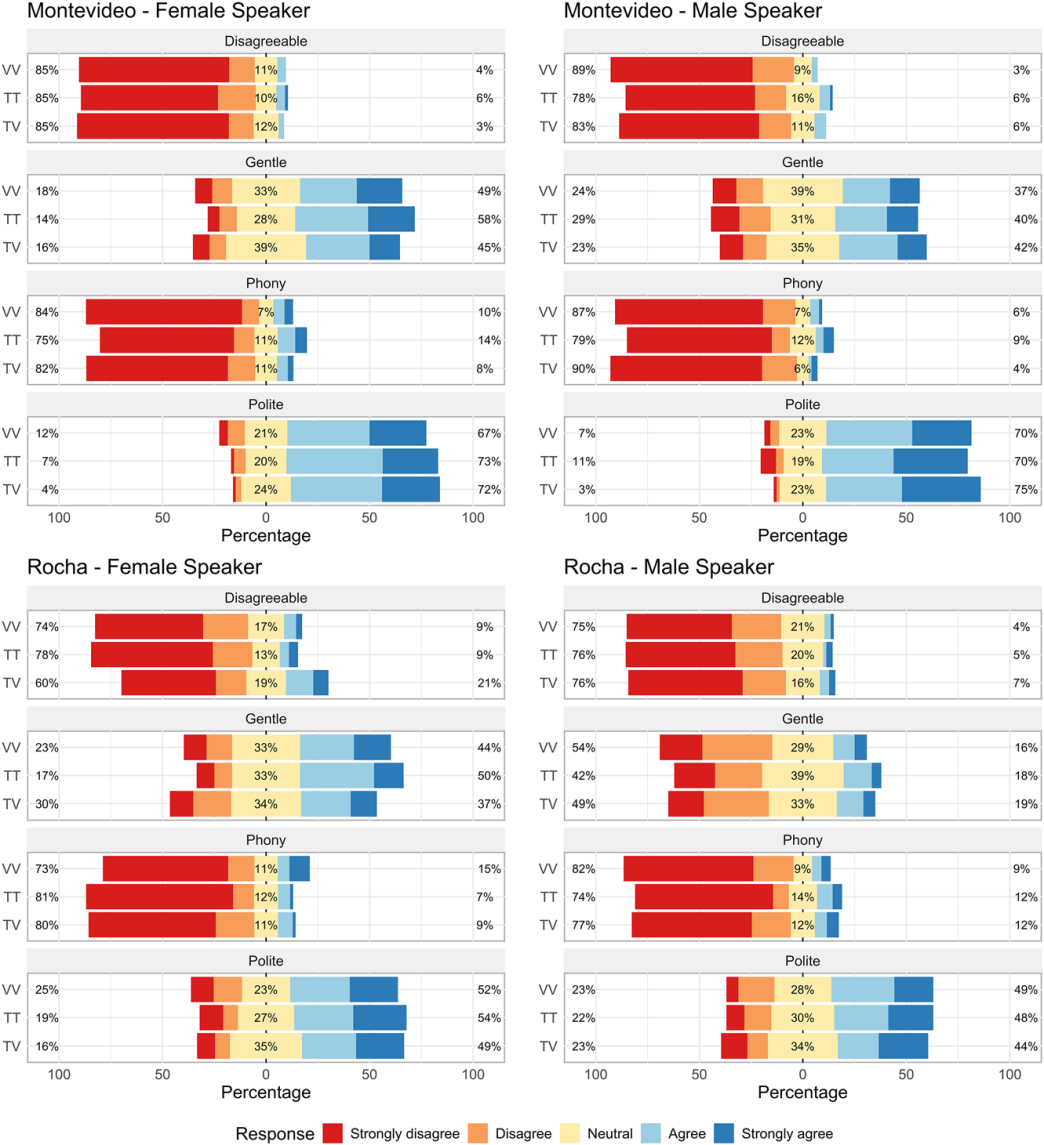
Distribution of the ratings on the solidarity traits of female and male guises across the three variants by participants in Montevideo and in Rocha.

Figure 5 displays the relative frequency of centered ratings (from “strongly disagree” to “strongly agree”) of Montevideo and Rocha participants on the solidarity traits across the three variants for each speaker. In both locations, participants generally perceived the speakers as polite (*cortés*) and gentle (*delicada*) and did not find them disagreeable (*antipática*) or phony (*falsa*). The female speaker was perceived as more gentle and more polite than the male speaker in both locations. Additionally, the male speaker was perceived as less gentle in Rocha compared to Montevideo.

The results from the fitted cumulative link mixed-effects models for each of the four solidarity traits are given in Table 5. In Montevideo, the VV variant was perceived as less phony than both TV and TT for the female speaker, while for the male speaker, the TV variant was perceived as less phony compared to the VV and TT variants. Across all three variants, the female speaker was considered phonier than the male speaker. On the other hand, in Rocha, participant gender was the only significant coefficient for the trait phony, suggesting that male raters perceived speakers as more phony compared to female raters. In Rocha, the female speaker was perceived as more disagreeable when using the TV variant compared to the TT and VV variants. For the male speaker, no significant difference was found across variants. In both locations, the male speaker was perceived as less gentle than the female speaker across the three variants.

**Table 5:** The results from the fitted cumulative link mixed-effects models for the solidarity traits. Reported are the coefficient estimates and their significance levels. Coefficients shown in boldface are statistically significant at the 0.05 level.

	Disagreeable		Gentle		Phony		Polite	
	Montevideo	Rocha	Montevideo	Rocha	Montevideo	Rocha	Montevideo	Rocha
VariantTT	0.45	−0.34	0.42	0.40	0.89***	−0.75	0.38	0.37
VariantTV	−0.34	0.71*	−0.15	−0.35	0.22***	−0.24	0.37	0.26
ReaderM	0.15	−0.12	−0.67*	−1.69***	−0.01***	−0.32	0.30	0.09
GenderM	0.72	0.83*	−0.54	−0.22	−0.24	1.18*	−0.38	0.74
Age	−0.09**	−0.03	0.06**	0.01	−0.08***	−0.02	0.05**	0.02
EducationNo	3.28***	0.38	0.59	−0.75	1.37*	−0.94	−1.11	−0.38
VariantTT × SpeakerM	0.13	0.26	−0.45	−0.11	−0.56***	0.82	−0.10	−0.34
VariantTV × SpeakerM	0.62	−0.63	0.36	0.60	−0.37***	0.59	0.22	−0.47
SD (Intercept ID)	2.98	1.28	2.17	1.69	2.38	1.58	2.15	1.89
Num.Obs.	440	403	440	414	441	412	441	419

Significance levels: *p < 0.05, **p < 0.01, ***p < 0.001.

The fitted models also suggest significant differences based on participant age and education level. In Montevideo, older participants perceived the speakers as less disagreeable, less phony, gentler, and more polite. By contrast, in Rocha, no significant association was found between age and perceptions of solidarity traits among participants. Additionally, in Montevideo, participants with higher education levels were more likely to disagree that the speakers were disagreeable and phony compared to those with lower education levels. In Rocha, however, no significant difference was found between the education groups regarding these perceptions. The relative positions of the variant-speaker gender combinations in each location can be found in Table C3 in Appendix – Part C.

Our use of a cumulative link mixed-effects model framework in the analysis of MGT data (as shown in Sections 4.1, 4.2, and 4.3), previously advocated in Guzzardo Tamargo et al. (2019) and further discussed in Loureiro-Rodríguez and Acar (2022), effectively incorporates repeated Likert-scale ratings from multiple respondents and facilitates the assessment of attitudinal differences towards guises, taking into account socio-demographic characteristics. By conducting separate analysis for each trait and location, we were able to identify unique location-specific attitudinal differences in each trait, which might have been overlooked if the data had been analyzed using factor analysis or principal component analysis.

## 5 Discussion

In what follows, we use the research questions that motivated this study as a way to organize and discuss our findings. We wrap up this section by considering the differences in speech community attitudes towards endogenous and exogenous variants when used by men and women and discuss how these differences may be linked to the dual prestige system discussed in Section 2.2.

### 5.1 Which variants (TT, VV, TV) are associated with Uruguayan identity, with Montevideo identity, and with Rocha identity?

First, we should note that all variants were perceived as Uruguayan in both locations. This suggests that most raters used phonetic and prosodic features as their main criteria for identifying the guises, rather than focusing narrowly on address forms. That said, these forms were also used as clues, in patterns that match dialectal variation across Uruguay. Thus, in Montevideo, the speakers were perceived as most Uruguayan in the VV condition, slightly less so when they used TV variants, and least so in their TT guises. By contrast, Rocha participants made no statistically significant differences in their evaluation of male guises (VV, TV, TT), but, interestingly, they thought the TT female guise was more Uruguayan than the other two. These differences by rater location and speaker gender suggest that in Montevideo raters’ definition of what it means to be Uruguayan was narrower along variants, since they conflated national identity with their own address form usage. These results are consistent with the responses of Montevideo female raters in Moyna and Loureiro-Rodríguez (2017). By contrast, in Rocha, identity definitions were more broadly construed for the male than for the female, since her Uruguayan identity was more closely tied to TT.

In both locations, speakers were perceived as less from Montevideo and more from Rocha when they used TT instead of VV or TV. Ratings also differed by speaker gender, which was unexpected. When using the VV variant, the female speaker was perceived as less from Rocha and more from Montevideo than the male speaker by both Montevideo and Rocha raters.

In other words, there was general agreement among raters that informal address pronouns are a distinctive feature that can help identify regional varieties. Thus, TT usage indexed Rocha identity for both Rocha and Montevideo raters, while Montevideo identity was identified by VV, and to a lesser extent, TV. This converging rating is expected if we consider the explicit assessment of *tuteo* as a positively valued feature of Rocha Spanish, not only among its users but more generally (Albertoni 2016; Weyers 2013; Weyers and Canale 2013). Interestingly, for the female speaker, identity was more closely linked to address form use than for the male, with the TT variant leading to higher ratings for Rocha identity and VV to higher ratings of Montevideo identity than for the corresponding male guises. This suggests that heightened attention is directed towards informal address variation in women than in men, a finding that we return to in Section 5.4.

### 5.2 Do participants from Rocha and Montevideo associate TT, VV, and TV with different status traits?

First, we should note that variation in address form had no impact on the evaluation of a speaker’s educational level, as represented by the trait *con estudios universitarios* ‘university educated.’ The similarity in the evaluation of this trait shows that in Uruguay no address variant is stigmatized or associated more strongly with lower socioeducational groups than others. Respondents from both locations likely use other cues to judge this attribute, such as phonetic and prosodic features, which remained invariable throughout the recordings. Since the two speakers were indeed matched in terms of their education and socioeconomic class, similarities in ratings are not surprising.

Let us now turn to the evaluation of the trait *correcta* ‘proper.’ Montevideo raters exhibited no differences in their ratings of variants for either the male or the female speaker. For their part, Rocha raters did not exhibit any statistically significant differences from Montevideo raters when it came to their evaluation of the male speaker. However, they did evaluate the female as significantly less proper when she used VV, in contrast with the TT and TV variants. This difference between the ratings of the male and the female speaker was unexpected and compatible with a higher level of scrutiny of address form use by women in Rocha. In other words, while Rocha raters were willing to give a pass to the male speaker shifting away from *tuteo* and towards *voseo*, a feature associated with a less “pure” national vernacular, not using the local variant came at a cost for the female speaker.

A similar pattern was found for the attribute *moderna* ‘modern’. Montevideo raters thought both speakers were less modern when using TT than when using TV, which in turn was rated as less modern than VV, as expected from prior studies of claimed usage in Montevideo (Weyers 2009), explicit attitudinal surveys (Weyers 2013; Weyers and Canale 2013), and the earlier implicit test with female raters (Moyna and Loureiro-Rodríguez 2017), all of which pointed to the association of *tuteo* with a prescriptive usage that has gradually become obsolete. It is also compatible with the evolution of informal address preferences in children’s literature and popular music, which have shifted from a prevalence of *tuteo* in the earlier periods (Moyna and Blumenthal 2023) to *voseo* in the new millennium (Moyna 2015; Rosales Lagos and Moyna 2016). By contrast, Rocha raters distinguished between the male and female speaker for the modern (*moderna*) trait: TV was rated as less modern than either TT or VV. This difference in rating is not only unexpected but puzzling in light of prior research. One might expect that both TV and VV would be associated with the national pattern and thus with change away from the local vernacular; however, this is not what the ratings point to. It might be that the respondents found the use of TV by a young voice jarring, given its associations with older women and the Montevideo upper class (Behares 1981; Bertolotti and Coll 2003; Weyers 2009). However, it is unclear why a similar effect was not reported for the male voice.

### 5.3 Do participants from Rocha and Montevideo associate TT, VV, and TV with different solidarity traits?

Of the four solidarity traits, only one, *antipática* ‘disagreeable’, showed rating differences by variant in Rocha. The pattern was similar to that of other attributes for that location: while the male speaker was not evaluated differently by variant, the female TV guise was rated as more disagreeable than either TT or VV. This rating parallels the more negative perception of the TV female guise, and more generally, is in keeping with the heightened scrutiny of address form choice by female speakers. Meanwhile, in Montevideo, only *falsa* ‘phony’ exhibited significant differences for both speakers. Both the male and the female were perceived as most phony in the TT guise; however, for VV and TV, their ratings were inverted. While she was perceived as least phony in the VV guise, he was thought to be least phony in the TV variant. While these results are complex and difficult to parse, we note they parallel previous findings where males were evaluated more favorably when using TV variants than their female counterparts (Moyna and Loureiro-Rodríguez 2017: 68).

### 5.4 Speech communities, gender, and prestige

The first overall observation from the ratings is that in some ways, raters from both locations behave as a single speech community, because they share perceptions of what the address forms mean. For example, Montevideo and Rocha raters gave similar ratings to identity traits, associating VV and TV with Montevideo, and TT with Rocha. In addition, for the most part rater characteristics such as education, age, and gender did not influence their attitudes towards identity differently.

One key difference between Rocha and Montevideo was the significance of speaker gender on the ratings of variants. In Rocha, the male speaker was consistently evaluated more leniently than the female when employing the non-local variants (TV, VV). By contrast, the female speaker seems to have been judged in terms of the extent to which she upheld local traits. No such difference was found in Montevideo.

This pattern suggests that raters in Rocha operate with three intersecting kinds of prestige. VV and TV hold global prestige (Eckert 1989), as forms associated with the capital city, national media, and educated urban speakers. In contrast, TT carries both covert and local prestige. It holds covert prestige through opposition to the national or capital-centered norms, and local prestige as a marker of membership in the community. TT in Rocha is seen not only as a symbol of local authenticity and solidarity, characteristics often linked to in-group identity and resistance to linguistic change (Labov 2001; Milroy and Milroy 1985), but also as normative and proper within the local context.

This complex prestige system may help explain the asymmetry observed in evaluations of male and female speakers. Men, particularly those in more peripheral communities, often enjoy greater flexibility to draw on forms associated with global or covert prestige without incurring social penalties; in contrast, women tend to be evaluated more closely in relation to local norms (Al-Essa 2009; Haeri 1991; Milroy and Milroy 1985). In Rocha, then, the female speaker’s use of non-local variants (VV and TV) may have conflicted with expectations tied to local identity, resulting in lower ratings. In Montevideo, by contrast, TT no longer carries global or covert prestige, since it is largely absent from everyday speech, and there is thus no expectation that speakers, either male or female, will employ them spontaneously. As a result, its use does not index either positive or negative traits, and speaker gender has no bearing on its perception. These findings underscore that prestige is not monolithic or stable; rather, it is socially situated and shaped by the intersection of linguistic form, speaker identity, and local ideologies of language (Eckert 2018).

## 6 Conclusions

This study employed a matched-guise test to examine how variation in informal address forms in Uruguayan Spanish (TT, VV, TV) is perceived in terms of local and national identity, status, and solidarity, by listeners from Montevideo and Rocha. Across both locations, TT was strongly associated with Rocha identity, while VV and, to a lesser extent, TV were linked to Montevideo. These patterns highlight the enduring role of informal address as a salient regional marker, even when other phonetic and prosodic features remain constant.

A key contribution from this study lies in revealing how speaker gender intersects with language attitudes. In Rocha, the female speaker’s use of address variants was judged more critically than the male speaker’s, which indicates that women – especially in smaller communities where local forms carry prestige – may be held to different linguistic standards than men. Overall, our findings demonstrate that VV and TV carry global prestige tied to the capital city, while TT embodies normative local prestige, covert prestige as a form of resistance to capital-centered norms, and local prestige as a marker of authenticity and solidarity.

Another important contribution concerns the methodological advantages and disadvantages of the MGT. The obvious advantage is that it expands the scope of our analysis beyond what language users themselves can explicitly articulate. Thus, while the attitudes about the identity value of informal address variants in USp are confirmed by using the MGT, the test repeatedly showed differences in how address variant use is evaluated in women compared to men, something heretofore unnoticed in any explicit test.

That said, there were also some inherent limitations in the recorded samples used in the test, because both speakers were in fact university-educated young adults from Montevideo. At the time of test design, this limitation was weighed against the possible effects of adding Rocha speakers, which would have lengthened the test, potentially causing fatigue and raising the number of incomplete responses. On the other hand, the absence of Rocha guises may have resulted in slightly lower Rocha identity ratings for the TT guises, which the Rocha raters may have considered less authentic than if they had been recorded by local speakers. This would be expected if Rocha raters were sensitive to phonetic or prosodic cues. Moreover, the ratings may also have been affected by dissonance between perceived speaker age and form use, in turn leading to lower ratings than expected for some guises (e.g., TV, which is probably better matched to older speakers [Bertolotti and Coll 2003; Weyers 2009]).

It may be possible in future studies to create guises with Rocha speakers using the three variants (VV, TV, TT), instead of or as well as Montevideo speakers, in order to mitigate the impact of phonetic cues on ratings. It may also be possible to repeat the test with older speakers, to see whether this results in different ratings. Other possible expansions could include raters from other cities, such as those from the western littoral of Uruguay along the Argentina border, which are much heavier *voseo* users, or from the department of Maldonado, which is recognized as a transitional area between Montevideo and Rocha. Given the higher levels of VV in the west, and the mixing of variants in Maldonado, it is very likely that different implicit attitudes will be identified.

## Appendix

### Part A: Texts

TT variant: Hola, Mati. No sabes lo que hice: me vine para la playa sin las reposeras. Por favor, tráelas tú cuando vengas el sábado. Si no las encuentras, llámame que te explico dónde están.

VV variant: Hola, Mati. No sabés lo que hice: me vine para la playa sin las reposeras. Por favor, traelas vos cuando vengas el sábado. Si no las encontrás, llamame que te explico dónde están.

TV variant: Hola, Mati. No sabés lo que hice: me vine para la playa sin las reposeras. Por favor, traelas tú cuando vengas el sábado. Si no las encontrás, llamame que te explico dónde están.

English: Hello, Mati. Guess what I did: I came to the beach without the beach chairs. Please, bring them with you when you come on Saturday. If you can’t find them, call me and I’ll tell you where they are.

### Part B: Survey question sample

**Table C1:** Relative positions of the variants by speaker gender with respect to VV-F for each identity trait and each location (Montevideo: MO, Rocha: RO). The variants showing no significant difference (after p-value adjustment with Tukey’s method) from VV-F are displayed in grey, while the ones identified as significantly different (at 5 % significance level after p-value adjustment) are given in bold, or black if borderline significant. For the latter cases, the corresponding p-values are reported in parentheses.

Trait	Location	− ←					VV-F					→ +
From MO	MO	**TT-F** (*p* < 0.0001)	**TT-M** (*p* < 0.0001)	**TV-M** (*p* < 0.0001)	**TV-F** (*p* < 0.0001)	**VV-M** (*p* = 0.0497)	VV-F					
	RO	**TT-F** (*p* < 0.0001)	**TT-M** (*p* < 0.0001)	**TV-M** (*p* = 0.0019)	**TV-F** (*p* = 0.0081)	VV-M	VV-F					
From RO	MO						VV-F	VV-M	**TV-F** (*p* = 0.0004)	**TV-M** (*p* < 0.0001)	**TT-M** (*p* < 0.0001)	**TT-F** (*p* < 0.0001)
	RO						VV-F	VV-M	**TV-F** (*p* = 0.0227)	**TV-M** (*p* = 0.0019)	**TT-M** (*p* < 0.0001)	**TT-F** (*p* < 0.0001)
Uruguayan	MO	**TT-M** (*p* = 0.0006)	**TT-F** (*p* = 0.0107)	**TV-M** (*p* = 0.0209)	TV-F	VV-M	VV-F					
	RO					TT-M	VV-F	TV-F	TV-M	VV-M	TT-F (*p* = 0.0696)	

**Table C2:** Relative positions of the variants by speaker gender with respect to VV-F for each status trait and each location (Montevideo: MO, Rocha: RO). The variants showing no significant difference (after p-value adjustment with Tukey’s method) from VV-F are displayed in grey, while the ones identified as significantly different (at 5 % significance level after p-value adjustment) are given in bold with the corresponding p-values given in parentheses.

Trait	Location	− ←					VV-F					→ +
Modern	MO	**TT-M** (*p* < 0.0001)	**TT-F** (*p* < 0.0001)	**TV-M** (*p* = 0.0006)	**VV-M** (*p* = 0.0049)	**TV-F** (*p* = 0.0388)	VV-F					
	RO	**TT-M** (*p* < 0.0001)	**TV-M** (*p* < 0.0001)	**TV-F** (*p* < 0.0001)	**VV-M** (*p* < 0.0001)	**TT-F** (*p* < 0.0001)	VV-F					
Proper	MO				VV-M	TT-F	VV-F	TV-F	TV-M	TT-M	TT-F	TT-M
	RO						VV-F	VV-M	TV-M	TV-F		
Higher educated	MO		TT-M	TT-F	TV-F	TT-M	VV-F	TV-M	VV-M			
	RO			TT-F	TV-F	TV-M	VV-F	VV-M				

**Table C3:** Relative positions of the variants by speaker gender with respect to VV-F for each solidarity trait and each location (Montevideo: MO, Rocha: RO). The variants showing no significant difference (after p-value adjustment with Tukey’s method) from VV-F are displayed in grey, while the ones identified as significantly different (at 5 % significance level after p-value adjustment) are given in bold with the corresponding p-values given in parentheses.

Trait	Location	− ←				VV-F					→ +
Disagreeable	MO	TT-F	TT-M	VV-M	TV-F	VV-F	VV-M	TV-M	TT-F	TT-M	
	RO				TV-M	VV-F	TV-F				
Gentle	MO	TT-M	VV-M	TV-M	TV-F	VV-F	TT-F				
	RO	**VV-M** (*p* < 0.0001)	**TV-M** (*p* = 0.0002)	**TT-M** (*p* = 0.0004)	TV-F	VV-F	TT-F				
Phony	MO	TT-F	VV-M	**TV-M** (*p* < 0.0001)	**VV-M** (*p* < 0.0001)	VV-F	**TV-F** (*p* < 0.0001)	**TT-M** (*p* < 0.0001)	**TT-F** (*p* < 0.0001)		
	RO			TT-M	TV-F	VV-F	TV-M				
Polite	MO				TV-M	VV-F	VV-M	TV-F	TT-F	TT-M	TV-M
	RO					VV-F	VV-M	TT-M	TV-F	TT-F	
